# Supplementary material for: Sex-specific transcriptomic responses to changes in the nutritional environment
Source: eLife. 2019 Aug 22;8:e47262. doi: 10.7554/eLife.47262 (PMC6773443; doi:10.7554/eLife.47262)
Supplement: Transparent reporting form [file elife-47262-transrepform.docx]

***eLife’s* transparent reporting form**

We encourage authors to provide detailed information *within their submission* to facilitate the interpretation and replication of experiments. Authors can upload supporting documentation to indicate the use of appropriate reporting guidelines for health-related research (see [EQUATOR Network](http://www.equator-network.org/%20)), life science research (see the [BioSharing Information Resource](https://biosharing.org/)), or the [ARRIVE guidelines](http://www.plosbiology.org/article/info:doi/10.1371/journal.pbio.1000412) for reporting work involving animal research. Where applicable, authors should refer to any relevant reporting standards documents in this form.

If you have any questions, please consult our Journal Policies and/or contact us: [editorial@elifesciences.org](mailto:editorial@elifesciences.org).

**Sample-size estimation**

- You should state whether an appropriate sample size was computed when the study was being designed
- You should state the statistical method of sample size computation and any required assumptions
- If no explicit power analysis was used, you should describe how you decided what sample (replicate) size (number) to use

Please outline where this information can be found within the submission (e.g., sections or figure legends), or explain why this information doesn’t apply to your submission:

We state our sample size in the methods section.

> Sample sizes for Experiment1 can be found in lines 194-196

In sum, 60 flies were used for each experimental treatment (2 sexes x 8 diets). This gives a total of 960 flies.

> Sample sizes for Experiment2 can be found in lines 286-287 + 299-307

In sum, we used 12 whole flies for each experimental treatment. Three independent biological replicates were used, giving a total of 12 libraries (2 sexes x 2 diets)

> Sample sizes for Experiment3 can be found in lines 360-362

In sum, 60 flies were used for each experimental treatment (2 sexes x 2 diets x 4 rapamycin)

**Replicates**

- You should report how often each experiment was performed
- You should include a definition of biological versus technical replication
- The data obtained should be provided and sufficient information should be provided to indicate the number of independent biological and/or technical replicates
- If you encountered any outliers, you should describe how these were handled
- Criteria for exclusion/inclusion of data should be clearly stated
- High-throughput sequence data should be uploaded before submission, with a private link for reviewers provided (these are available from both GEO and ArrayExpress)

Please outline where this information can be found within the submission (e.g., sections or figure legends), or explain why this information doesn’t apply to your submission:

Replicate information can be found alongside sample size.

We state our replicates size in the methods section.

> Replicate sizes for Experiment1 can be found in lines 194-196

In summary, 20 biological replicates were used for each experimental treatment (sex/diet combination)

> Replicate sizes for Experiment2 can be found in lines 286-287 + 299-307

In summary, 3 biological replicates were used for each RNASeq library (for all treatments)

> Replicate sizes for Experiment3 can be found in lines 360-362

In summary, 20 biological replicates were used for each experimental treatment (sex/diet/rapamycin combination)

**Statistical reporting**

- Statistical analysis methods should be described and justified
- Raw data should be presented in figures whenever informative to do so (typically when N per group is less than 10)
- For each experiment, you should identify the statistical tests used, exact values of N, definitions of center, methods of multiple test correction, and dispersion and precision measures (e.g., mean, median, SD, SEM, confidence intervals; and, for the major substantive results, a measure of effect size (e.g., Pearson's r, Cohen's d)
- Report exact p-values wherever possible alongside the summary statistics and 95% confidence intervals. These should be reported for all key questions and not only when the p-value is less than 0.05.

Please outline where this information can be found within the submission (e.g., sections or figure legends), or explain why this information doesn’t apply to your submission:

We have a dedicated statistical analysis section at the end of every experiment where we justify the statistics used for our study.

We report p-values plus associated statistic (F & correlation coefficients). If p-values are below 0.001, then we report as p<0.001. If p-values are above this number, then we report exact p-value. In addition, we report instances where p-value is above 0.05.

(For large datasets, or papers with a very large number of statistical tests, you may upload a single table file with tests, Ns, etc., with reference to sections in the manuscript.)

**Group allocation**

- Indicate how samples were allocated into experimental groups (in the case of clinical studies, please specify allocation to treatment method); if randomization was used, please also state if restricted randomization was applied
- Indicate if masking was used during group allocation, data collection and/or data analysis

Please outline where this information can be found within the submission (e.g., sections or figure legends), or explain why this information doesn’t apply to your submission:

Flies were randomly allocated to groups. This information can be found on the first section of each experiment.

**Additional data files (“source data”)**

- We encourage you to upload relevant additional data files, such as numerical data that are represented as a graph in a figure, or as a summary table
- Where provided, these should be in the most useful format, and they can be uploaded as “Source data” files linked to a main figure or table
- Include model definition files including the full list of parameters used
- Include code used for data analysis (e.g., R, MatLab)
- Avoid stating that data files are “available upon request”

Please indicate the figures or tables for which source data files have been provided:

Datafiles used to make the plots can be found in Supplementary files provided with this submission.
